# Finite element analysis of wedge and biconcave deformity in four different height restoration after augmentation of osteoporotic vertebral compression fractures

**DOI:** 10.1186/s13018-021-02225-8

**Published:** 2021-02-15

**Authors:** Xiao-Hua Zuo, Yin-Bing Chen, Peng Xie, Wen-Dong Zhang, Xiang-Yun Xue, Qian-Xi Zhang, Ben Shan, Xiao-Bing Zhang, Hong-Guang Bao, Yan-Na Si

**Affiliations:** 1 Department of Anesthesiology, Nanjing First Hospital, Nanjing Medical University, Nanjing, 210006 China; 2 Department of Pain Management, The Affiliated Huai’an Hospital of Xuzhou Medical University and The Second People’s Hospital of Huai’an, Huai’an, 223002 China; 3 Department of Orthopedic Surgery, The Affiliated Haian Hospital of Nantong University, Haian, 226600 China; 4 Department of Neurosurgery, The Affiliated Huai’an Hospital of Xuzhou Medical University and The Second People’s Hospital of Huai’an, Huai’an, 223002 China; 5 Department of Orthopedics, Northern Jiangsu People’s Hospital, Yangzhou, 225001 China; 6 Department of Pain Management, Yancheng No.1 People’s Hospital, Yancheng, 224000 China; 7 Department of Radiology, The Affiliated Huai’an Hospital of Xuzhou Medical University and The Second People’s Hospital of Huai’an, Huai’an, 223002 China

**Keywords:** Osteoporotic vertebral compression fractures, Percutaneous vertebral augmentation, Finite element analysis, Anterior wedge deformity, Biconcave deformity

## Abstract

**Purpose:**

Biomechanical comparison of wedge and biconcave deformity of different height restoration after augmentation of osteoporotic vertebral compression fractures was analyzed by three-dimensional finite element analysis (FEA).

**Methods:**

Three-dimensional finite element model (FEM) of T11-L2 segment was constructed from CT scan of elderly osteoporosis patient. The von Mises stresses of vertebrae, intervertebral disc, facet joints, displacement, and range of motion (ROM) of wedge and biconcave deformity were compared at four different heights (Genant 0–3 grade) after T12 vertebral augmentation.

**Results:**

In wedge deformity, the stress of T12 decreased as the vertebral height in neutral position, flexion, extension, and left axial rotation, whereas increased sharply in bending at Genant 0; L1 and L2 decreased in all positions excluding flexion of L2, and T11 increased in neutral position, flexion, extension, and right axial rotation at Genant 0. No significant changes in biconcave deformity. The stress of T11-T12, T12-L1, and L1-L2 intervertebral disc gradually increased or decreased under other positions in wedge fracture, whereas L1-L2 no significant change in biconcave fracture. The utmost overall facet joint stress is at Genant 3, whereas there is no significant change under the same position in biconcave fracture. The displacement and ROM of the wedge fracture had ups and downs, while a decline in all positions excluding extension in biconcave fracture.

**Conclusions:**

The vertebral restoration height after augmentation to Genant 0 affects the von Mises stress, displacement, and ROM in wedge deformity, which may increase the risk of fracture, whereas restored or not in biconcave deformity.

## Introduction

Osteoporotic vertebral compression fractures (OVCFs) are among the most common complications of osteoporosis, a systemic bone disorder with a decline in bone mineral density and degradation skeletal microarchitecture [1–3]. In the population over 65 years and older, the prevalence of OVCFs is more than 30% and increases with age. OVCFs commonly afflict most elderly population resulting in pain, spinal deformity, functional incapacity, decreased quality of life, and increased mortality. OVCFs have become a more progressively severe disease and a significant health problem worldwide that would apparently increase social and economic burdens to society and family [4–8].

Progressive spinal deformity occurred, subsequent to sagittal imbalance, overload of facet joints, paraspinal muscle spasm, and occasional impingement of sympathetic nerve or spinal nerve [9, 10]. Melton et al. divided the vertebral fracture’s morphology into three types: wedge, biconcave, and crush fracture [11]. The difference in fracture type is most likely due to differences in biomechanical effects [12]. Wedge fracture is the loss of the height of the anterior vertebral, which can lead to the change of kyphosis angle, forward center of gravity, and the sagittal imbalance [13, 14]. The biconcave fracture does not affect the kyphosis. It can directly affect the upper and lower endplates and the intervertebral disc, affecting the stress distribution. Although the crush fracture does not affect the kyphosis, it affects the stress distribution and the collapse of the posterior vertebrae resulting in reduced intervertebral foramen. Percutaneous vertebral augmentation (PVA) is a treatment for strengthening the fractured vertebrae and restoring the height of the vertebrae [15, 16]. In patients with OVCFs, the fractured vertebral height can be completely, partially, or not restored after vertebral augmentation. The fractured vertebral collapses again after PVA in some patients, which may be related to the stress unevenness after vertebral augmentation.

In 1974, Belytschko et al. first applied FEA for biomechanical analysis in the spine [17]. At present, FEA is widely used in mechanics of spinal diseases [18–21]. Researchers have analyzed the effects of different vertebral height of OVCFs on the von Mises stress of vertebral bodies after PVA in wedge fracture [22]. Currently, little research regarding biomechanical effects of different types of vertebral deformity has been reported with von Mises stresses, displacement, and ROM by FEA. Vertebral deformity of OVCFs results in the mechanical instability of the vertebrae. Different types of fractures of OVCFs may have different biomechanics patterns. Therefore, we hypothesized that there are biomechanical differences in the recovery of vertebrae from different fracture types (wedge and biconcave fractures) in OVCFs. The present study aimed to explore the biomechanical effects between two different types of vertebral deformity with von Mises stresses, displacement, and ROM by three-dimensional (3D) FEA. By analyzing the differences of T12 wedge and biconcave deformity in four different height restoration (Genant 3, 2, 1, 0) after augmentation of OVCFs, we will determine the consistency of biomechanical results and fracture type with different vertebral height for deciding which height restoration must be performed.

## Methods

### The FEA models of T11-L2 segment in elderly osteoporosis

A 65-year-old female osteoporosis patient was selected without other abnormal findings on radiographs. After signing the informed consent, the geometry of the thoracolumbar spine was reconstructed from 0.5-mm thick and intervals computerized tomography (CT) scans of the patient. CT scan images were processed with Mimics 16.0 (Materialise, Leuven, Belgium) and transformed into a solid model. After repair, denoise and spheroidality with Geomagic Studio 2014 (Geomagic, Morrisville, USA), and assemble with Pro/E5.0 (PTC, MA, USA), the T11-L2 thoracolumbar geometry model was then imported into the Hypermesh13.0 (Altair, California, USA) for meshing. Finite element meshing secondary processing, other tissue meshing, and analyzed were performed in MSC.Patran/Nastran2012 (MSC, USA). The FEM construct comprised vertebrae, posterior elements (including cortical and cancellous bone), intervertebral discs, endplates, anterior longitudinal ligament (ALL), posterior longitudinal ligament (PLL), capsular ligament (CL), intertransverse ligament, ligamentum flavam (ITL), interspinous ligament (ISL), and supraspinous ligament (SSL). The four-node tetrahedral elements modeled in vertebrae and eight-node hexahedral elements intervertebral disc. The ligaments were assumed to be two-node nonlinear spring elements subjected only to tensile load. The surface-surface contact elements were applied to simulate facet joints, and the coefficient of friction was assigned to 0.2 [23].

### FEM validation

The validation of the established FEM is crucial for utilizing a model to simulate authentic responses. The ROM data were in comparison with the results of a cadaveric biomechanical investigation [24–26] that applied a similar load (10N·M moment, 150N), in flexion, extension, lateral bending (left and right), and axial rotation (left and right). The FEM was validated because of the calculated ROM approximate to the literature, which indicated that the model could be implemented in the following application of OVCFs.

### The FEA models of T11-L2 segment in OVCFs

**Fig. 1:**
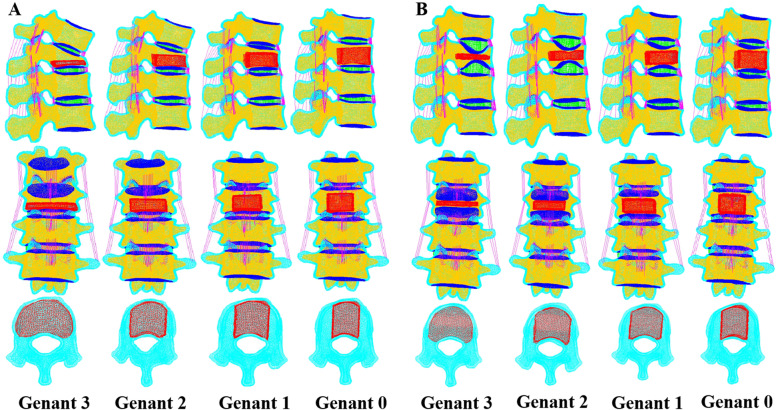
A schematic showing height and cement location of wedge fracture (**a**) and biconcave fracture (**b**)

According to Genant semi-quantitative method (0–3 grade), Genant 0–3 of the T12 anterior wedge deformity and biconcave deformity were constructed respectively. Different vertebral heights of T12 vertebral body after vertebral augmentation were simulated as Genant grade. The simulation of cement filling was executed based on the knowledge related to the clinical prescription of vertebral augmentation. The geometry and distribution of the cement filling were defined as the previous study. Bone cement equivalent to 30% of vertebral volume was filled to the trabecular of T12 vertebra. Material properties of finite element analysis models are shown in Table 1 [19, 23]. Figure 1 illustrates a schematic showing the location, shape, and overall size of the bone cement in the vertebrae.

**Table 1:** Material properties of finite element analysis models

Component	Young Modulus(MPa)	Poisson ratio	Cross-sectional area(mm^2^)
Cortical bone	8040	0.30	--
cancellous bone	37	0.20	--
Bony end-plate	670	0.40	--
Posterior structure	2345	0.25	--
Fibrous annulus	4.2	0.45	--
Nucleus pulposus	0.2	0.49	--
Annulus matrix	500	0.30	--
Cement	3000	0.40	--
Articular facet cartilage	10	0.40	--
Anterior longitudinal ligament	20	0.30	57
Posterior longitudinal ligament	70	0.30	24
Supraspinous ligament	28	0.30	8
Interspinous ligament	28	0.30	28
Capsular ligament	20	0.30	48
Ligamentum flavum	50	0.30	49
Intertransverse ligament	50	0.30	9

### Loading and boundary conditions

The multi-level FEM of T11-L2 was used for analysis. The lower surface of the L2 endplate was characterized to be rigidly anchored, and the loads were implemented on the upper surface of the T11 endplate. An axial compressive preload of 500N was set, and a torsional moment of 10 N·M was dictated to imitate the motions of neutral position (NP), flexion (FLEX), extension (EXT), left lateral bending (LLB), right lateral bending (RLB), left axial rotation (LAR), and right axial rotation (RAR) [18, 27–29]. The overall displacement and ROM of T11-L2 segment in four heights were investigated and compared with the two types of fracture. The peak von Mises stress on vertebrae, intervertebral disc, facet joint, and the stress distribution was also used for analysis.

## Results

### Comparative analysis of von Mises stress

**Fig. 2:**
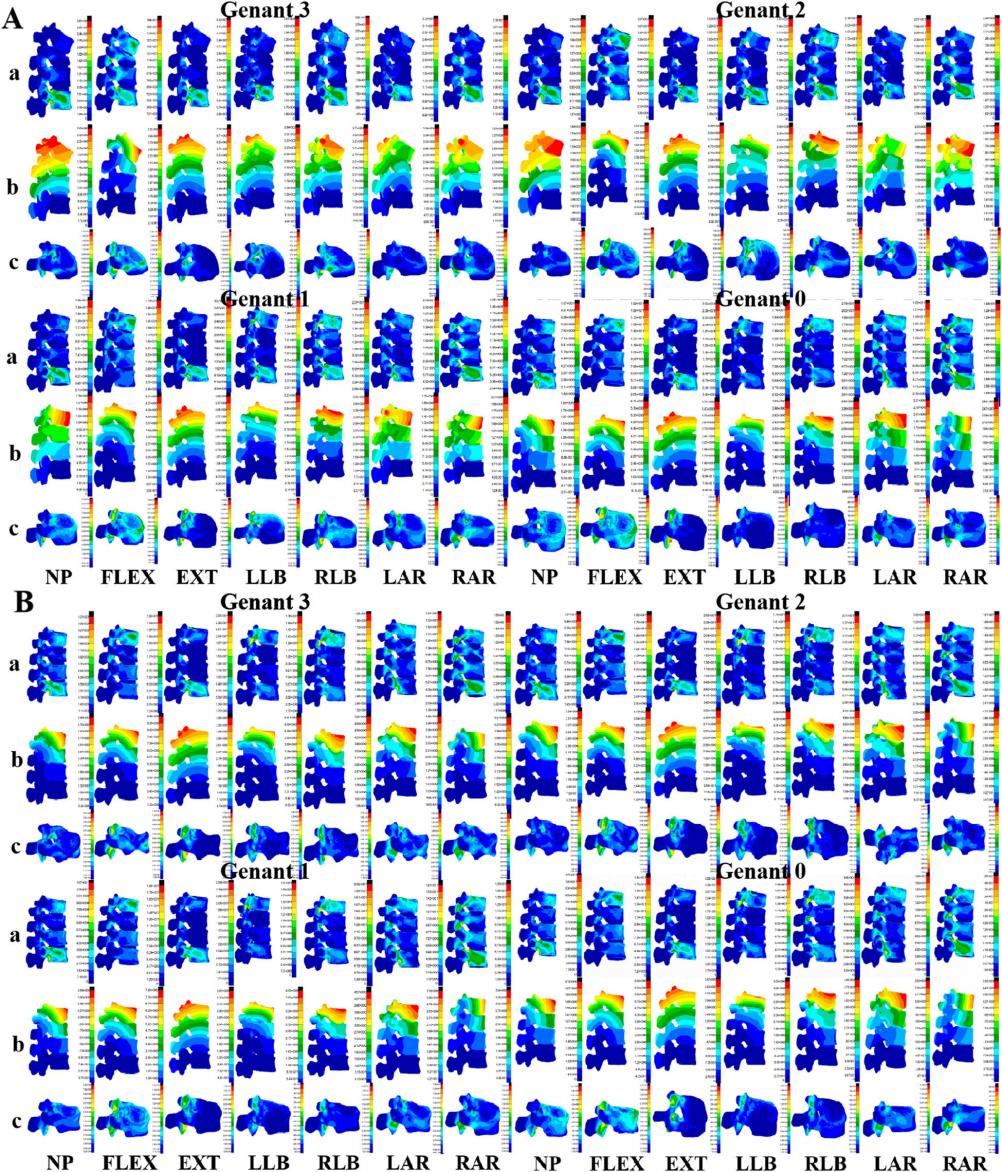
Nephograms of the von Mises stress (MPa) and displacement (mm) after different vertebral restoration of T12 osteoporotic vertebral compression fractures of wedge fracture (**a**) and biconcave fracture (**b**). Note: The overall vertebral stress (**a**), displacement (**b**), and T12 vertebral stress (**c**), neutral position (NP), flexion (FLEX), extension (EXT), left lateral bending (LLB), right lateral bending (RLB), left axial rotation (LAR), and right axial rotation (RAR)

**Fig. 3:**
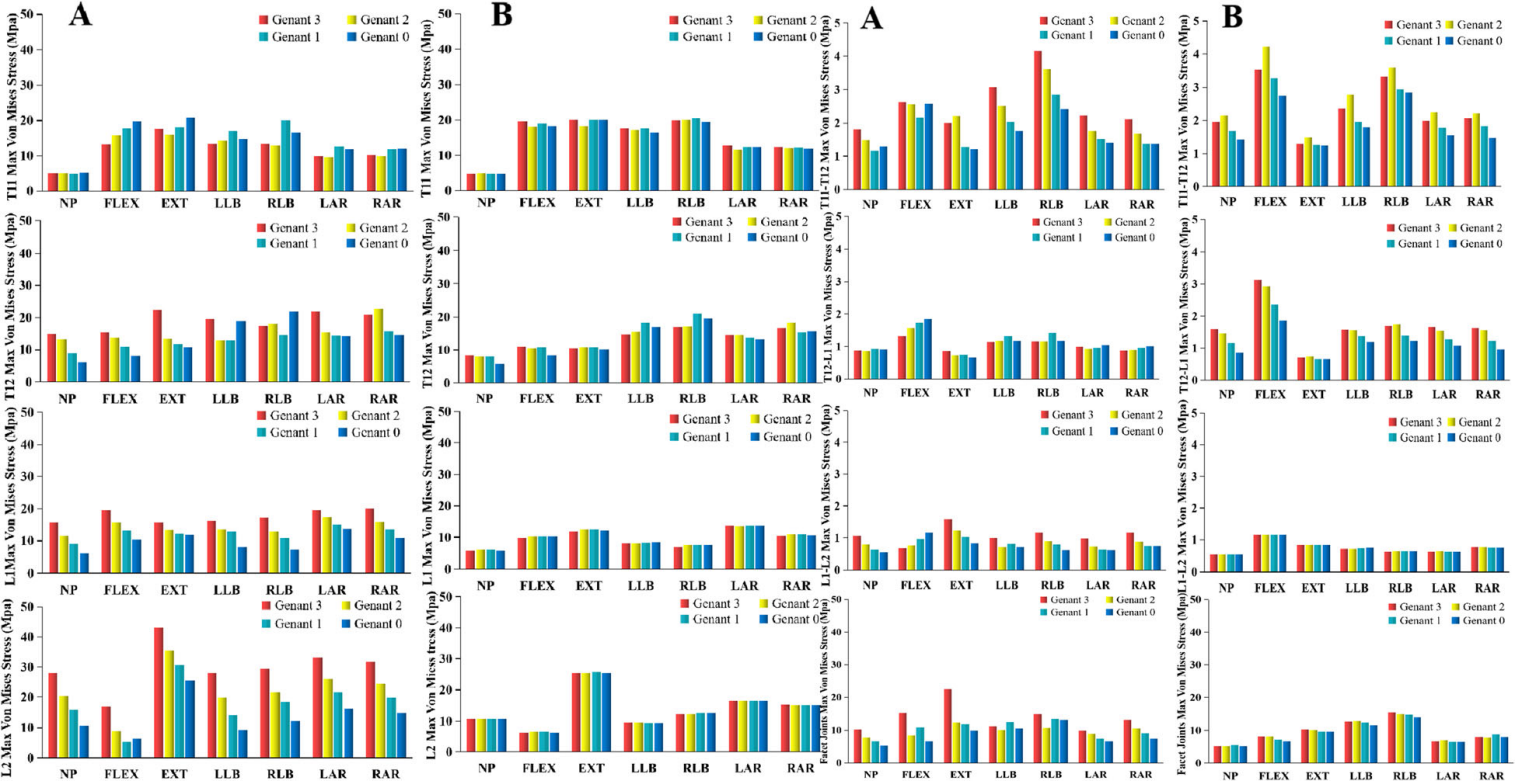
The von Mises stress of the vertebral body, intervertebral disc, and facet joint under seven different postures of wedge fracture (**a**) and biconcave fracture (**b**). Note: neutral position (NP), flexion (FLEX), extension (EXT), left lateral bending (LLB), right lateral bending (RLB), left axial rotation (LAR), and right axial rotation (RAR)

The nephograms of the overall vertebral stress, displacement, and T12 vertebral stress are shown in Fig. 2. The von Mises stress peaks of the vertebrae intervertebral disc and facet joint shown in Fig. 3. Wedge deformity in the position of neutral position, flexion, extension, and left axial rotation, the von Mises stress of T12 decreased as the restoration height of vertebrae to Genant 3, 2, 1, and 0, respectively, whereas increased sharply at Genant 0 in lateral bending. The von Mises stress of adjacent vertebrae (L1 and L2) decreased slowly with the height of vertebrae in all positions excluding flexion of L2, and the stress of T11 vertebrae was increased in some positions and decreased in other positions. The stress of T11, T12, L1, and L2 vertebrae did not significantly change with the increase of recovery height in biconcave deformity.

The stress of T11-T12 intervertebral disc decreased gradually with the height in lateral bending and axial rotation position in wedge fracture, but there is no noticeable change trend in biconcave deformity. The stress of T12-L1 intervertebral disc was gradually increased with the height of vertebrae when flexion in wedge fracture, while the stress was similar under seven positions in biconcave fracture. The stress gradually decreases with the height under seven positions in biconcave fracture. The stress of L1-L2 intervertebral disc gradually increased with the height of the vertebral body under flexion in wedge fracture and decreased under neutral position, extension, right lateral bending, and axial rotation. There was no significant change with vertebral height under the same position in biconcave fracture.

The overall facet joint stress was gradually decreased in wedge fracture, and the most considerable decreased stress in flexion and the utmost stress is in all positions at Genant 3. There was no significant change in the overall facet joint stress in the same position of biconcave fracture.

### Comparative analysis of displacement and ROM

**Fig. 4:**
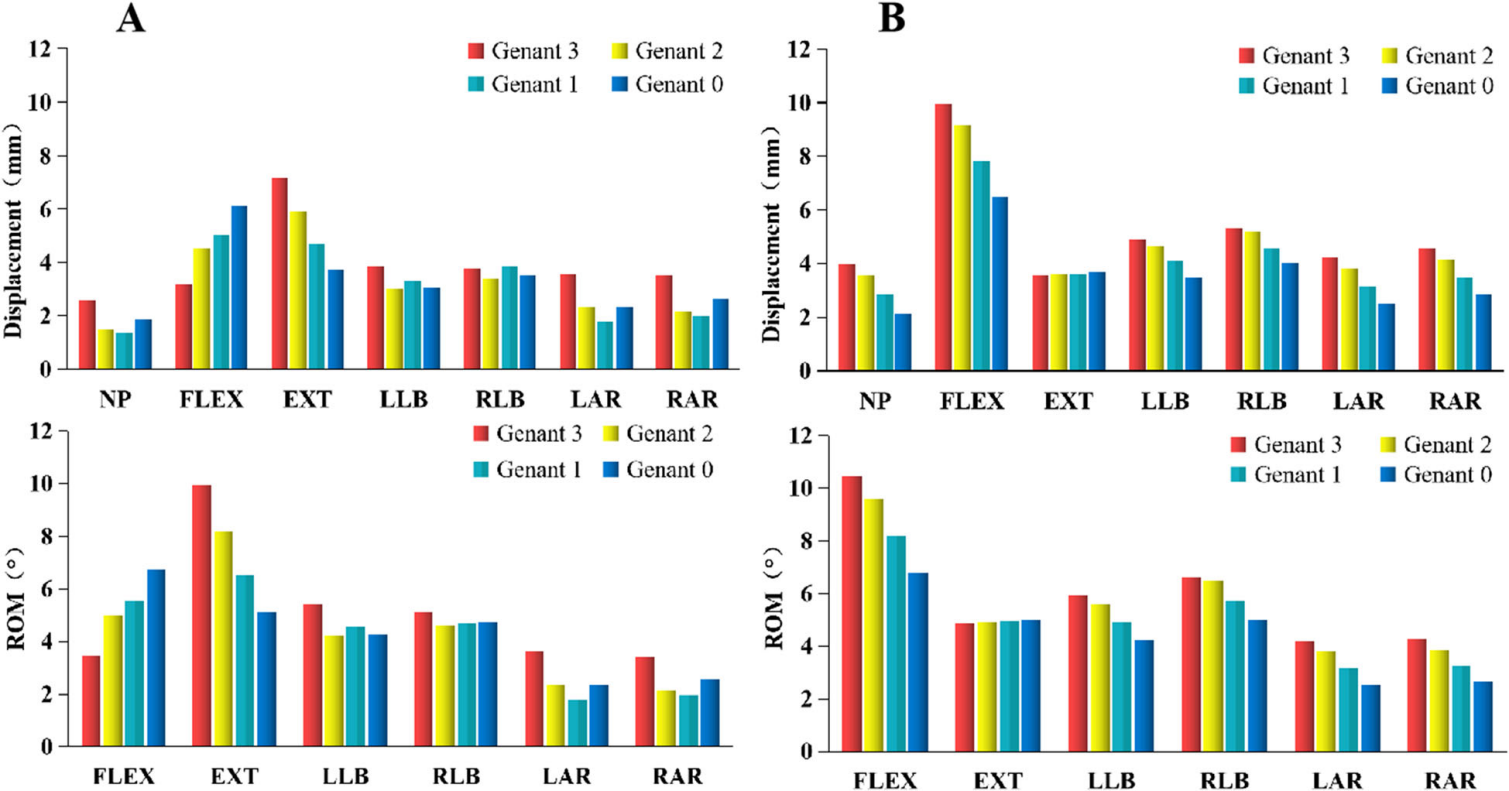
Displacement and ROM corresponding to Genant classification under seven different postures of wedge fracture (**a**) and biconcave fracture (**b**). Note: neutral position (NP), flexion (FLEX), extension (EXT), left lateral bending (LLB), right lateral bending (RLB), left axial rotation (LAR), and right axial rotation (RAR)

The nephograms of displacement are shown in Fig. 2b. The displacement and ROM showed in Fig. 4. In wedge fracture, the overall displacement and ROM of the T11-L2 vertebrae gradually increased with the vertebral height in flexion and decreased in extension, while the trend was opposite in the biconcave fracture. The displacement and ROM increased with the vertebral height, and the maximum is about 94% at Genant 0 in contrast to Genant 3 in flexion, while the extension stretched gradually, the displacement and ROM decreased most 48% at Genant 0 in the wedge fracture. The displacement and ROM increased slowly under extension in the biconcave fracture and decreased gradually in the other six positions. The displacement and ROM of the wedge fracture have increased and decreased, while decreased slowly in all positions excluding extension in biconcave fracture.

## Discussion

In this study, elderly osteoporosis patient was selected, and CT data were obtained. The FEM of osteoporosis T11-L2 was successfully established and validated. Based on the above, we research on one of the frequent thoracolumbar spine fractures (T12), one of the suitable stiffness bone cement volume (equivalent to 30% of T12 vertebral volume), and top two fracture type (wedge and biconcave) of OVCFs [30–32]. We demonstrated whether different fracture type (wedge and biconcave) has a potentially effect on height restoration after augmentation of OVCFs. To clarify the biomechanical comparison of wedge and biconcave deformity of different height restoration after augmentation of OVCFs, we performed finite element analysis of the von Mises stresses of vertebrae, intervertebral disc, facet joints, displacement, and range of motion (ROM) of wedge and biconcave deformity were compared at four different heights (Genant 3, 2, 1, 0) after T12 vertebral augmentation. Collectively, our data demonstrated that the vertebral height after vertebral augmentation restores to Genant 0 in wedge deformity, affecting the von Mises stress, displacement, and ROM, which may increase the risk of fracture, whereas restoration height restored or not in biconcave deformity.

The vertebral morphology of OVCFs varies in shape and height. Melton et al. divided the spine fracture into three types: wedge deformity, biconcave deformity, and crush deformities [11]. The Genant visual semi-quantitative method is based on the reduction in the anterior, middle, and posterior vertebral heights by lateral X-rays. It has been widely used in clinical and epidemiological studies [3, 33–35]. OVCF patients with wedge fractures after PVA recovery of anterior vertebral can moderately improve kyphosis and center of gravity forward for restoration in sagittal imbalance and reduce the stress on the paraspinal muscles of patients with OVCFs to maintain balance [36]. Although the biconcave deformities after PVA do not affect the kyphosis and the center of gravity, it can improve the biomechanical changes of the endplates of the fractured vertebral and adjacent intervertebral discs and influence the mechanical distribution of adjacent vertebral. In this study, the stress of T12 in wedge deformity decreased as the vertebral height in neutral position, flexion, extension, and left axial rotation, whereas increased sharply in bending at Genant 0 and no significant changes in biconcave deformity. The displacement and ROM of the wedge fracture had ups and downs, while a decline in all positions excluding extension in biconcave fracture.

Some researchers have analyzed the biomechanical of the L2 fractured vertebral of OVCFs after PVA by finite element analysis, which can enhance the strength of the fractured vertebral and increase the vertebral load. In the study of patients with OVCFs, bone cements (3, 5, and 10 ml) with different elastic moduli (1800, 500, and 150 MPa) were injected into the L2 fractured vertebrae. In the neutral position, flexion and extension, lateral bending and axial rotation position, the vertebral showed similar maximum von Mises stress, and the maximum stress of the cortical bone and the lower endplate adjacent to the vertebral body L1 increased significantly. In the neutral position, flexion, extension, and lateral bending (left and right) positions, the maximum von Mises stress of the vertebral increases with the increase of the elastic modulus of the cement [37]. The finite element analysis of patients with OVCFs after PVA of T12 fractured vertebral suggests that insufficient bone cement and asymmetric distribution may lead to a maximum displacement of the vertebral body, and a significant increase in maximum von Mises stress of cancellous and cortical bone, which may lead to re-fracture of the T12 vertebral and fracture of the adjacent vertebral [38]. This finite element analysis did not study the different heights of OVCFs after PVA. Based on the FEM of T12 wedge deformity in patients with non-osteoporosis, the maximum von Mises stress of the lower endplate of T11 vertebral and the upper endplate of L1 vertebrae, and the compression of T12 anterior vertebral (compressed to 90%, 80%, 70%, 60%, 50%, 40%, 30%, 20%, and 10%) are positively correlated [39]. There was no significant difference between the maximum von Mises stresses of vertebral heights (Genant 3, 1, and 0) after T12 undifferentiated fracture typed OVCFs augmentation [22]. However, unlike the previous research, our study compared wedge and biconcave deformity. This difference in fracture type may account for the different result.

## Conclusion

The vertebral height after vertebral augmentation restores to Genant 0 in wedge deformity, affecting the von Mises stress, displacement, and ROM, which may increase the risk of fracture, whereas restoration height restored or not in biconcave deformity.

## Data Availability

The datasets used and/or analyzed during the current study are available from the corresponding author on reasonable request.

## Abbreviations

FEA: Finite element analysis
FEM: Finite element model
OVCFs: Osteoporotic vertebral compression fractures
PVA: Percutaneous vertebral augmentation
ROM: Range of motion
CT: Computerized tomography
